# Preventive effect of heat-killed *Lactiplantibacillus plantarum* FM8 in an ovalbumin-induced food allergy murine model

**DOI:** 10.1186/s13223-026-01048-8

**Published:** 2026-06-25

**Authors:** Hideaki Takahashi, Tadashi Fujii, Chikako Yamada, Kotoyo Fujiki, Kento Kuramitsu, Shoei Okuda, Mamoru Tanaka, Takayuki Asahina, Kohei Funasaka, Eizaburo Ohno, Yoshiki Hirooka, Takumi Tochio

**Affiliations:** 1 Graduate School of Nutritional Sciences, Nagoya University of Arts and Sciences, Nisshin, Aichi 470-0196 Japan; 2 Department of Gastroenterology and Hepatology, Fujita Health University, Toyoake, Aichi 470-1192 Japan; 3 BIOSIS Lab. Co., Ltd, Toyoake, Aichi 470-1192 Japan; 4 Department of Medical Research on Prebiotics and Probiotics, Fujita Health University, Toyoake, Aichi 470-1192 Japan; 5 Department of Applied Biosciences, Graduate School of Bioagricultural Sciences, Nagoya University, Nagoya, Aichi 464-8601 Japan; 6 Graduate School of Bioscience and Biotechnology, Chubu University, 1200 Matsumoto, Kasugai, Aichi 487-8501 Japan; 7 College of Bioscience and Biotechnology, Chubu University, 1200 Matsumoto, Kasugai, Aichi 487-8501 Japan; 8 Minori. Co., Ltd., Okayama, 701-1221 Japan

**Keywords:** Food allergy, Paraprobiotics, *Lactobacillus plantarum* FM8, Gut microbiome, Ovalbumin, Interleukin-10

## Abstract

**Background:**

Paraprobiotics, the non-viable microbial cells with health benefits, have gained interest as candidates for preventing food allergies owing to their safety and stability. Heat-killed *Lactiplantibacillus plantarum* FM8 (FM8) has been reported to induce interleukin (IL)-10 production in dendritic cells in vitro; however, its in vivo efficacy as a standalone intervention remains unexplored. We investigated the preventive effect of heat-killed FM8 in a murine model of ovalbumin (OVA)-induced food allergy.

**Methods:**

BALB/c mice were assigned to control, OVA-induced allergy, or three FM8 treatment groups receiving FM8 at low (2 × 10⁹ CFU/day), medium (1 × 10¹⁰ CFU/day), or high (5 × 10¹⁰ CFU/day) doses. Food allergy was induced by repeated OVA sensitization and challenge. Allergy symptoms, OVA-specific immunoglobulin E (IgE), and IL-10 levels were measured. Tight junction gene expression, mucin production, gut microbiota composition, and short-chain fatty acids (SCFAs) were also analyzed.

**Results:**

FM8 supplementation attenuated allergic responses in a dose-dependent manner. Allergy symptom scores were reduced in the High group, and the rectal temperature decline following OVA challenge was ameliorated in both the Med and High groups. The sensitization-induced increase in OVA-specific IgE was also attenuated in these groups. *IL-4* expression in Peyer’s patches was reduced, whereas colonic *IL-10* expression and serum IL-10 levels were increased in the High group. FM8 supplementation was further associated with increased ileal expression of tight junction-related genes (*Occludin*,* Claudin-1* and *Zo-1*) and *Muc2*, along with a trend toward increased fecal mucin levels; however, no comparable changes in tight junction-related gene expression were observed in the colon. Although taxa with reported SCFA-producing potential were enriched in the High group, cecal acetate and propionate levels remained unchanged, while butyrate levels were decreased.

**Conclusions:**

FM8 may prevent food allergy by promoting IL-10-associated immunoregulation and enhancing gut barrier-related responses, without a corresponding increase in cecal SCFA concentrations. The contribution of SCFA-related mechanisms remains to be clarified, and further studies are needed to determine clinically feasible dosing and efficacy in humans.

**Graphical abstract:**

**Figure:**
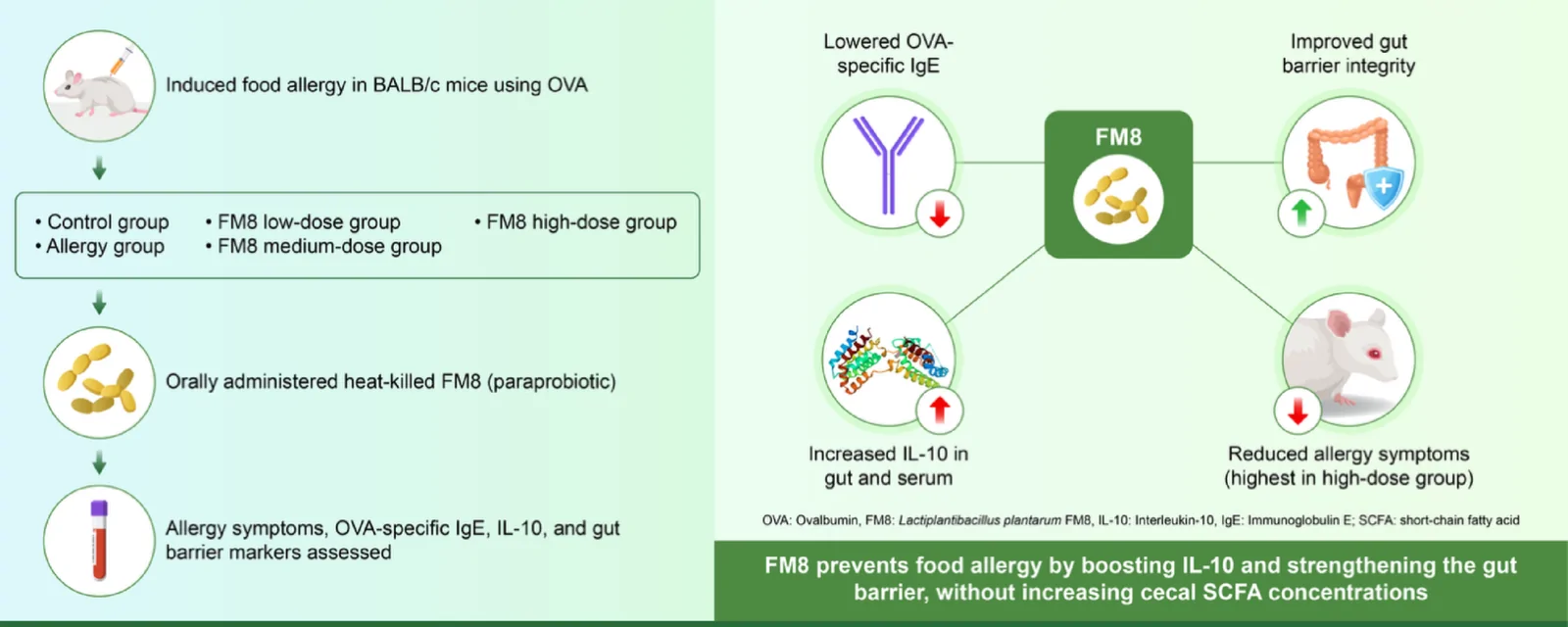

**Supplementary Information:**

The online version contains supplementary material available at https://doi.org/10.1186/s13223-026-01048-8.

## Background

Food allergies are defined as “adverse health effects arising from specific immune responses that occur reproducibly upon exposure to a given food” [1]. The prevalence of food allergy has increased dramatically in recent decades [2], representing a major public health concern. Current management strategies, including allergen avoidance and oral immunotherapy, impose substantial burdens on patients and their families, contributing to reduced quality of life and significant financial costs [3–5]. These challenges highlight the urgent need for novel preventive strategies targeting the underlying immunological mechanisms.

The pathogenesis of food allergy is primarily driven by T helper type 2 (Th2) cells, which promote B-cell-mediated immunoglobulin E (IgE) production [6, 7]. IgE antibodies recognize specific food allergens and bind to high-affinity IgE receptors on mast cells, triggering the release of inflammatory mediators such as histamine [6, 7]. This process can compromise epithelial tight junction integrity, increasing intestinal permeability and allergen uptake [8]. Conversely, interleukin-10 (IL-10) is an anti-inflammatory cytokine that modulates immune homeostasis by suppressing Th2 responses and promoting regulatory T (Treg) cell differentiation, thereby inhibiting IgE class switching and mitigating allergic reactions [9, 10].

Recent evidence has underscored the critical role of the gut microbiota in immune regulation and its association with susceptibility to allergic disease [11]. Probiotics, defined as “live microorganisms, which when administered in adequate amounts, exert a beneficial effect on the host’s health” [12], have gained attention as potential modulators of gut microbiota composition. Probiotics stimulate gut-associated immune cells and induce IL-10 production, thereby attenuating allergic responses [13, 14]. However, the use of live microorganisms presents challenges related to safety, storage, and stability, necessitating the exploration of alternative strategies [15, 16].

In this context, the concept of paraprobiotics has recently emerged. Paraprobiotics are defined as “non-viable microbial cells (either intact or broken), or crude cell extracts, which, when administered (orally or topically) in adequate amounts, confer a benefit on the human or animal consumer” [17]. Owing to their non-viable nature, paraprobiotics offer advantages such as improved safety and stability, as they eliminate risks associated with uncontrolled bacterial proliferation while retaining bioactivity [18]. Their ease of incorporation into food products further enhances their potential applicability. To date, research on paraprobiotics has primarily focused on inflammatory bowel disease [19, 20], where they have demonstrated effects comparable to those of probiotics [21, 22]. However, their potential in preventing food allergies remains underexplored.

A previous study demonstrated that heat-killed *Lactiplantibacillus plantarum* FM8, originally isolated from pickled vegetables, induces IL-10 production in vitro in cultured dendritic cells [23]. Parasynbiotics refer to approaches combining a paraprobiotic with a prebiotic, which is defined as “a substrate that is selectively utilized by host microorganisms conferring a health benefit” [24]. A parasynbiotic formulation combining FM8 with the prebiotic 1-kestose has been reported to exert anti-inflammatory effects in penguins [23] and to improve feline atopic skin syndrome [25]. However, these effects have only been demonstrated in vitro or in the context of parasynbiotics, whereas the in vivo efficacy of FM8 as a standalone intervention remains unexplored.

Therefore, in this study, we aimed to evaluate the efficacy of heat-killed *Lactiplantibacillus plantarum* FM8 against food allergy in an ovalbumin (OVA)-induced mouse model.

## Methods

### Mice

Overall, 40 female BALB/c mice (5 weeks old; 15–20 g) were purchased from SLC Japan (Hamamatsu, Japan). The mice were housed individually in cages lined with soft chips under standard laboratory conditions (24 ± 2 °C; 12-h light/dark cycle; adequate air flow) with ad libitum access to food and water. Experimental diets in powdered form were obtained from CLEA Japan (Tokyo, Japan). To minimize environmental bias, cages were placed randomly within the facility. All animal experiments were conducted in accordance with the ARRIVE guidelines and the Animal Experimentation Guidelines of our institute. The study protocol was approved by the Laboratory Animal Care Committee of the university, in compliance with the standards of the Ministry of the Environment (approval no. 155).

### Preparation of OVA

OVA was prepared following previously described methods [26]. Briefly, OVA used for mouse sensitization was isolated and purified from commercial egg whites using ammonium sulfate salting-out followed by isoelectric precipitation with sulfuric acid. OVA was confirmed by the presence of a single band on sodium dodecyl sulfate-polyacrylamide gel electrophoresis.

### Experimental design, development of OVA-induced murine model of food allergy, and sample collection

**Fig. 1:**
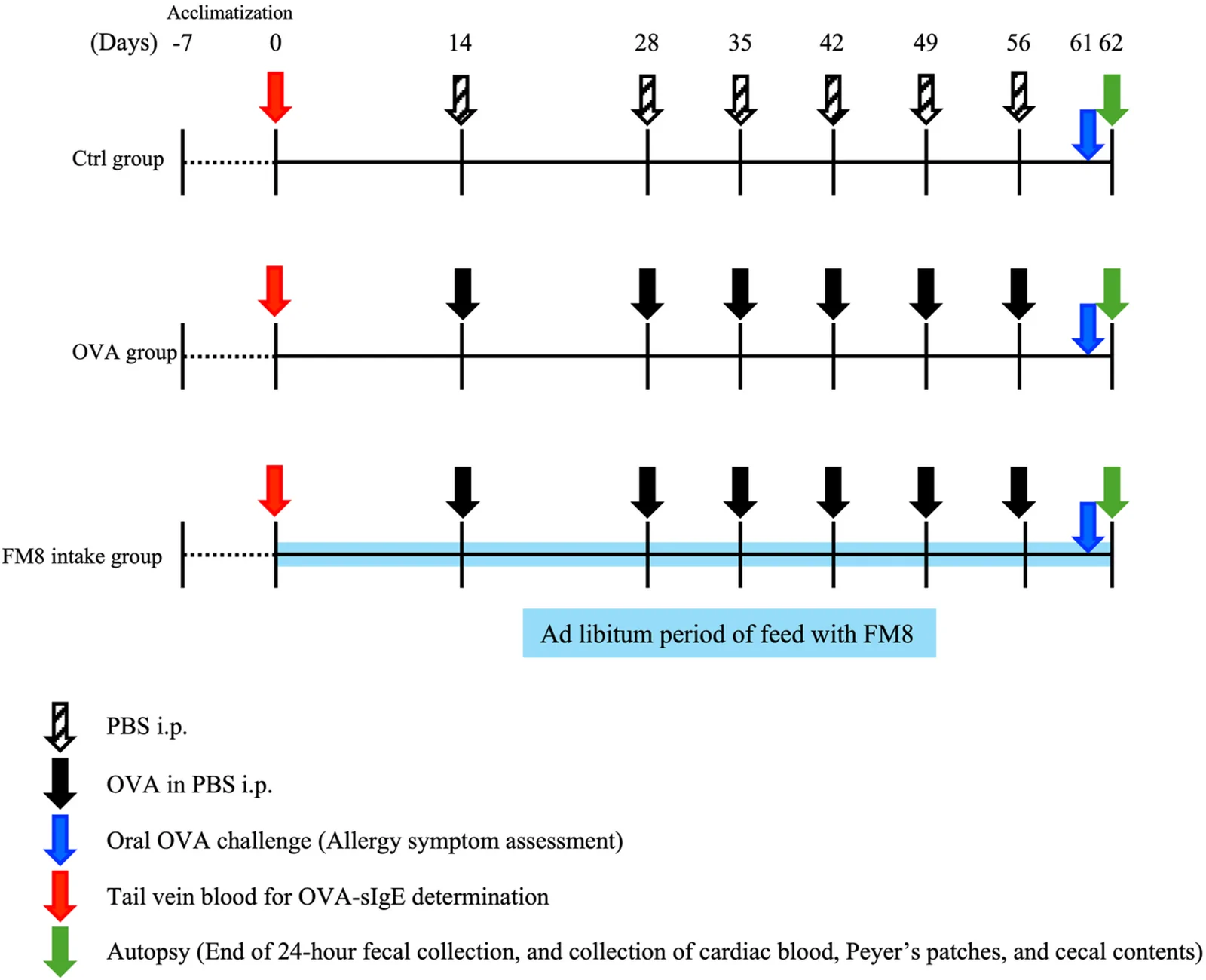
Experimental schedule for establishing the ovalbumin-induced food allergy mouse model

Figure 1 illustrates the experimental design. Food allergy was induced using an established BALB/c mouse model, commonly used in allergy research, sensitized via intraperitoneal administration [27]. Mice were randomly assigned into five groups—each comprising eight mice—using Excel: control (Ctrl), OVA-induced allergy (OVA), OVA-induced allergy with FM8 low dose (Low), OVA-induced allergy with medium dose FM8 (Med), and OVA-induced allergy with high dose FM8 (High). After 1 week of acclimatization on an AIN-93G semi-synthetic diet (CLEA Japan), experimental diets were administered from day 0 (before OVA sensitization) until autopsy to evaluate primary prevention of OVA allergy. Owing to dropouts, the final group sizes were Ctrl (*n* = 8), OVA (*n* = 8), Low (*n* = 7), Med (*n* = 8), and High (*n* = 7). All diets were based on AIN-93G; experimental diets contained heat-killed *L. plantarum* FM8 (MINORI Inc., Tokyo, Japan; 4.0 × 10^11^ CFU/g) at doses calculated according to average feed intake to achieve daily FM8 intakes of 2 × 10^9^ CFU (Low), 1 × 10^10^ CFU (Med), and 5 × 10^10^ CFU (High). The FM8 dose range (2 × 10^9^, 1 × 10^10^, and 5 × 10^10^ CFU/day) were selected to evaluate dose dependency across an exploratory range based on prior animal studies using heat-killed FM8 [23, 25]. Mice were immunized intraperitoneally with OVA six times between days 14 and 56. Ctrl mice received phosphate-buffered saline (PBS) injections. After the final OVA challenge, allergic symptoms were assessed, and fecal samples were collected. The following day, mice were anaesthetized with isoflurane, and anesthesia was confirmed by loss of the righting reflex and absence of pain responses to toe, tail, and ear stimulation with tweezers. Cardiac puncture was then performed as a method of euthanasia, and blood was collected for measurement of IgE and IL-10 levels. Peyer’s patches, ileum, and colon were harvested for *IL-10* mRNA expression analysis. Additional ileum and colon samples were collected for histological staining. Cecal contents were harvested for microbiota analysis and short-chain fatty acids (SCFAs) quantification. Serum samples were stored at − 20 °C, while cecal contents and tissues were stored at − 80 °C until further analysis.

### Induction of food allergy in mice

Food allergy was induced based on established protocols [26]. On day 14, mice in all groups except the Ctrl group were intraperitoneally immunized with 100 µg of OVA dissolved in PBS combined with 100 µL of aluminum hydroxide. Subsequent booster immunizations were performed on days 28, 35, 42, 49, and 56, each consisting of 50 µg of OVA with 50 µL of aluminum hydroxide in PBS. Mice in the Ctrl group received intraperitoneal injections of aluminum hydroxide in PBS alone at the corresponding time points.

### Evaluation of allergic symptoms

Allergic symptoms and rectal temperature changes were evaluated using previously described methods [26]. On day 61, all groups were orally challenged with 50 mg of OVA dissolved in 200 µL of PBS. Post-challenge, allergy scores were assessed for 30 min by multiple blinded researchers, and the maximum score during this period was recorded. Scoring criteria were as follows: 0, no symptoms; 1, scratching around the nose and head with abnormal behavior; 2, swelling around the eyes and mouth; 3, wheezing, difficult respiration, or cyanosis around the mouth and tail; 4, lack of activity upon stimulation, tremors, or convulsions; and 5, death [28]. Rectal temperature was measured using a thermometer immediately before and 30 min after oral administration of OVA, and the temperature change was calculated. The humane endpoint was defined as a ≥ 3 °C decrease in rectal temperature; however, no mice met this criterion during the experiment.

### Evaluation of OVA-specific IgE (OVA-sIgE) and IL-10

OVA-sIgE levels were measured following a previously described method with minor modifications [26]. Briefly, 96-well microplates (Nunc-Immuno Plate; Thermo Fisher Scientific, Waltham, MA, USA) were coated overnight at 4 °C with OVA solution (1 mg/mL in PBS). After blocking with 1% skim milk in PBS containing 0.05% Tween-20 at 37 °C for 1 h, serum samples diluted 1:9 in PBS were added and incubated at 37 °C for 1 h. Subsequently, POD-conjugated anti-mouse IgE antibody (GAM/IgE (Fc)/PO; Nordic Immunology Laboratories, Tilburg, Netherlands) diluted 1:1,000 in blocking buffer was added, followed by incubation at 37 °C for 1 h. Color development was performed using tetramethylbenzidine, and the reaction was terminated by adding 1 N HCl. Absorbance was measured at 450 nm using a microplate reader (Corona Electric, Hitachinaka, Japan).

Serum IL-10 levels were measured using Mouse IL-10 ELISA Kit (#Cat No. KE10008) according to the manufacturer’s instructions (Proteintech, Rosemont, IL, USA). Absorbance was measured at 450 nm with correction at 630 nm using a microplate reader (Corona Electric). Serum samples were diluted 1:1 with the provided diluent.

### RNA extraction and quantitative reverse transcription PCR (RT-qPCR)

RNA extraction, reverse transcription, and quantitative PCR were performed following previously reported methods [26]. Briefly, RNA was extracted from Peyer’s patches, ileum, and colon samples using a BioMasher II Micro Tissue Homogenizer (DWK Life Sciences, Millville, NJ, USA) and the RNeasy Mini Kit (Qiagen, Hilden, Germany). RNA concentrations were measured using the Qubit™ RNA Broad-Range Assay Kit (Invitrogen, Carlsbad, CA, USA). Reverse transcription was conducted with the High-Capacity RNA-to-cDNA Kit (Thermo Fisher Scientific). RT-qPCR was performed using the QuantStudio 3 Real-Time PCR System (Thermo Fisher Scientific) with PowerTrack SYBR Green Master Mix (Thermo Fisher Scientific) according to the manufacturer’s instructions. Sequences of the primers targeting *IL-10*,* Muc2*,* Occludin*,* Claudin-1*, and *Zo-1*, with 18S rRNA serving as the reference gene, are provided in eTable S1 [29–31]. The amplification protocol included an initial hold at 95 °C for 2 min, followed by 40 cycles of 15 s at 95 °C and 60 s at 60 °C. A melting curve analysis was performed post-amplification to confirm product specificity. Gene expression levels were calculated using the ΔΔCt method.

### Histology

Histological analyses were performed as previously described [32]. Briefly, ileum and colon samples were fixed in 4% paraformaldehyde phosphate buffer solution for 7 days and embedded in paraffin. Sections of 4-µm thickness were prepared and stained with periodic acid-Schiff (PAS)-Alcian blue (AB) for goblet cell enumeration. Stained tissue sections were observed and imaged using an upright microscope (Nikon Eclipse Ti-U; Nikon Co., Tokyo, Japan) equipped with NIS Elements software (Ver. 4.30).

### Analyses of fecal mucin

Mucin assays were performed as previously described [32]. Briefly, mucins were quantified using a fluorometric assay kit for fecal mucin analysis (Cosmo Bio Co., Ltd., Tokyo, Japan). Fluorescence intensity was measured at an excitation wavelength of 336 nm and an emission wavelength of 383 nm using a microplate reader (POWERSCAN® HT; BioTek Instruments, Winooski, VT, USA).

### 16S rRNA gene sequencing

Genomic DNA was extracted from cecal samples following previously reported methods [33]. Frozen cecal samples were thawed on ice, and 100 mg of each sample was suspended in a solution containing 4 M guanidium thiocyanate, 100 mM Tris-HCl (pH 9.0), and 40 mM ethylenediaminetetraacetic acid. Samples were disrupted using zirconia beads in a FastPrep FP100A instrument (MP Biomedicals, Santa Ana, CA, USA). DNA was extracted from the bead-treated suspensions using the Magtration System 12GC and GC series MagDEA DNA 200 kit (Precision System Science, Matsudo, Japan). For PCR amplification of the V3–V4 region of prokaryotic 16S rRNA genes, Pro341F and Pro805R primers were used (eTable S1). Sequencing was conducted at the Bioengineering Lab (Sagamihara, Japan). Paired-end sequencing (2 × 300 bp) was performed using the Illumina MiSeq platform (Illumina, San Diego, CA, USA) with a MiSeq Reagent Kit v3. The NGS datasets were deposited in the NCBI Sequence Read Archive under accession numbers PRJNA1298223.

### Bioinformatics analysis

QIIME2 (v2023.5) was used for 16S rRNA gene sequence analysis [34]. Quality filtering and denoising were performed using the DADA2 pipeline with parameters set to p-trunc-len-f 240 and p-trunc-len-r 240 [35]. Filtered sequences were assigned to taxa using the “qiime feature-classifier classify-sklearn” command with default settings [36], referencing the Greengenes2 database (v2022.10) for taxonomic assignment [37]. Alpha diversity was calculated, and principal coordinate analysis (PCoA) was conducted using weighted UniFrac distances via the “qiime diversity core-metrics-phylogenetic” command. Beta diversity was assessed using weighted UniFrac distances and the “qiime diversity beta-group-significance” command.

### Determination of SCFAs and lactate concentrations in cecal content samples

The quantities of SCFAs including acetate, propionate, and butyrate in cecal samples were analyzed following the methods in previous report [26]. Briefly, the carboxyl groups of SCFAs in each cecal sample were labeled with 2-nitrophenyl hydrazide using a Short- and Long-Chain Fatty Acid Analysis Kit (Cat# XSSFACR01, YMC, Kyoto, Japan) according to the improved kit protocol.

### Statistical analyses

All data are presented as mean ± standard error of the mean. Statistical analyses were performed using GraphPad Prism (version 10.4.2; GraphPad Software). Comparisons between the Ctrl and OVA groups were conducted using the Mann–Whitney U test. Comparisons between the OVA group and FM8 intervention groups were performed using the Kruskal–Wallis test followed by Dunn’s multiple comparisons test. Statistical significance was set at *p* < 0.05, and a trend was defined as *p* < 0.1.

## Results

### Baseline characteristics

No significant differences were observed in body weight or feed intake among the groups during the study period, indicating that neither OVA sensitization nor FM8 supplementation adversely affected general health status. FM8 was incorporated into the diets at doses designed to achieve daily intake levels of approximately 2 × 10^9^, 1 × 10^10^, and 5 × 10^10^ CFU in the Low, Med, and High groups, respectively, with actual intake closely matching these targets. Additionally, the cecal weight-to-body weight ratio and the cecal content weight-to-body weight ratio at the time of sacrifice did not differ among the groups (eTable S2), confirming consistent physiological conditions across all groups.

### Effect of FM8 on OVA food allergy markers

**Fig. 2:**
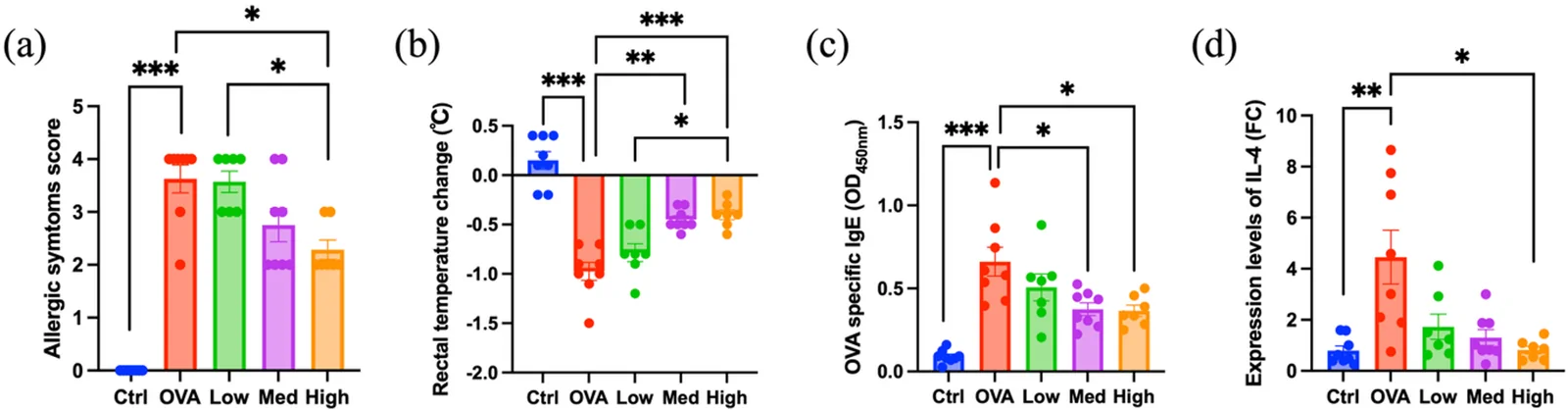
Severity of ovalbumin (OVA)-induced food allergy with and without FM8 administration. (**a**) Allergy scores in BALB/c mice. (**b**) Changes in rectal temperature 30 min after oral administration of OVA. (**c**) OVA-specific IgE levels in serum. (**d**) *IL-4* gene expression levels in Peyer’s patches. Plots represent individual mice, and bars represent mean ± standard error of the mean. *p < 0.05, **p < 0.01, ***p < 0.001 vs. OVA group (Mann–Whitney U test or Kruskal–Wallis test followed by Dunn’s multiple comparisons test). Control (Ctrl): non-allergy group, no intervention; OVA: OVA-induced allergy group, no intervention; Low: OVA allergy group administered FM8 at a low dose. Med: OVA allergy group administered FM8 at a medium dose. High: OVA allergy group administered FM8 at a high dose

To assess the efficacy of FM8 in preventing food allergy symptoms, allergy scores and rectal temperature changes were evaluated following oral OVA challenge. The OVA group exhibited significantly higher allergy scores than those of the Ctrl group (*p* = 0.0002), confirming allergic responses. The High group showed significantly lower allergy scores, compared with those of the OVA group (*p* = 0.0165), and a significant difference was observed between the Low- and High groups (*p* = 0.0358; Fig. 2a). Additionally, rectal temperature, a physiological marker known to decrease during allergic reactions, was significantly lower in the OVA group than in the Ctrl group (*p* = 0.0002). This reduction was significantly ameliorated in both the Med- and High groups (*p* = 0.0039 and 0.0009, respectively), with a significant difference detected between the Low- and High groups (*p* = 0.0421; Fig. 2b). Furthermore, evaluation of serum levels of OVA-sIgE before and after sensitization revealed no significant differences among the groups before sensitization (eTable S2). However, following sensitization, the OVA group displayed a significant increase in OVA-sIgE levels, compared with those of the Ctrl group (*p* = 0.0002). This increase was significantly attenuated in the Med- and High groups (*p* = 0.0419 and 0.0344, respectively; Fig. 2c). Moreover, *IL-4* expression levels in Peyer’s patches were significantly elevated in the OVA group (*p* = 0.0011), whereas this elevation was significantly suppressed in the High group (*p* = 0.0089; Fig. 2d). These findings suggest that FM8 exerts a protective, dose-dependent effect against OVA-induced food allergy.

### Effects of FM8 on IL-10 mRNA expression in gut tissue and serum IL-10

**Fig. 3:**
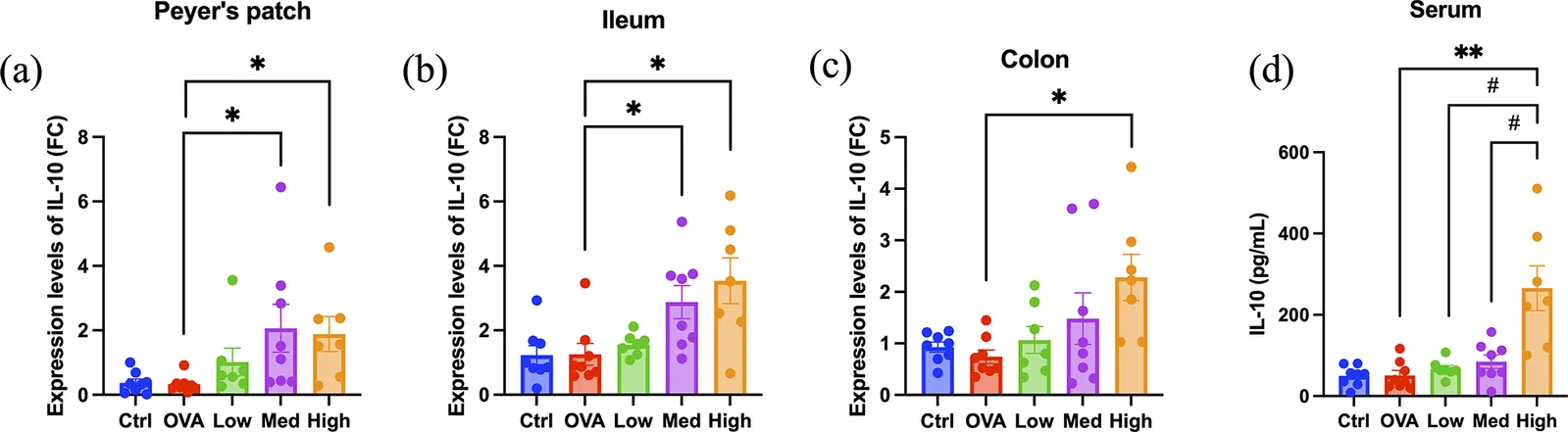
IL-10 levels in tissues and serum following FM8 supplementation. Expression levels of *IL-10* in (**a**) Peyer’s patches, (**b**) ileum, and (**c**) colon, and (**d**) IL-10 concentrations in serum. Plots represent individual mice, and bars represent mean ± standard error of the mean. #*p* < 0.1, *p < 0.05, **p < 0.01 vs. ovalbumin (OVA) group (Mann–Whitney U test or Kruskal–Wallis test followed by Dunn’s multiple comparisons test). Control (Ctrl): non-allergy group, no intervention. OVA: OVA-induced allergy group, no intervention. Low: OVA allergy group administered FM8 at a low dose. Med: OVA allergy group administered FM8 at a medium dose. High: OVA allergy group administered FM8 at a high dose

FM8 induces IL-10 production in vitro [23]. Therefore, we evaluated the effects of FM8 on IL-10 expression in gastrointestinal tissues and serum. *IL-10* mRNA expression was significantly increased in Peyer’s patches and the ileum in the Med and High groups compared with that of the OVA group (Peyer’s patches: *p* = 0.0143 and 0.0178; ileum: *p* = 0.0384 and 0.0157; Fig. 3a, b). In the colon, a significant increase in *IL-10* mRNA expression was observed only in the High group (*p* = 0.0297; Fig. 3c). Consistently, serum IL-10 levels were significantly elevated in the High group compared with those in the OVA group (*p* = 0.0013). Among the treatment groups, serum IL-10 levels showed a dose-dependent increasing trend, with marginal differences among the groups (Low vs. High, *p* = 0.0541; Med vs. High, *p* = 0.0820; Fig. 3d).

### Effects of FM8 on gut homeostasis and mucin production

**Fig. 4:**
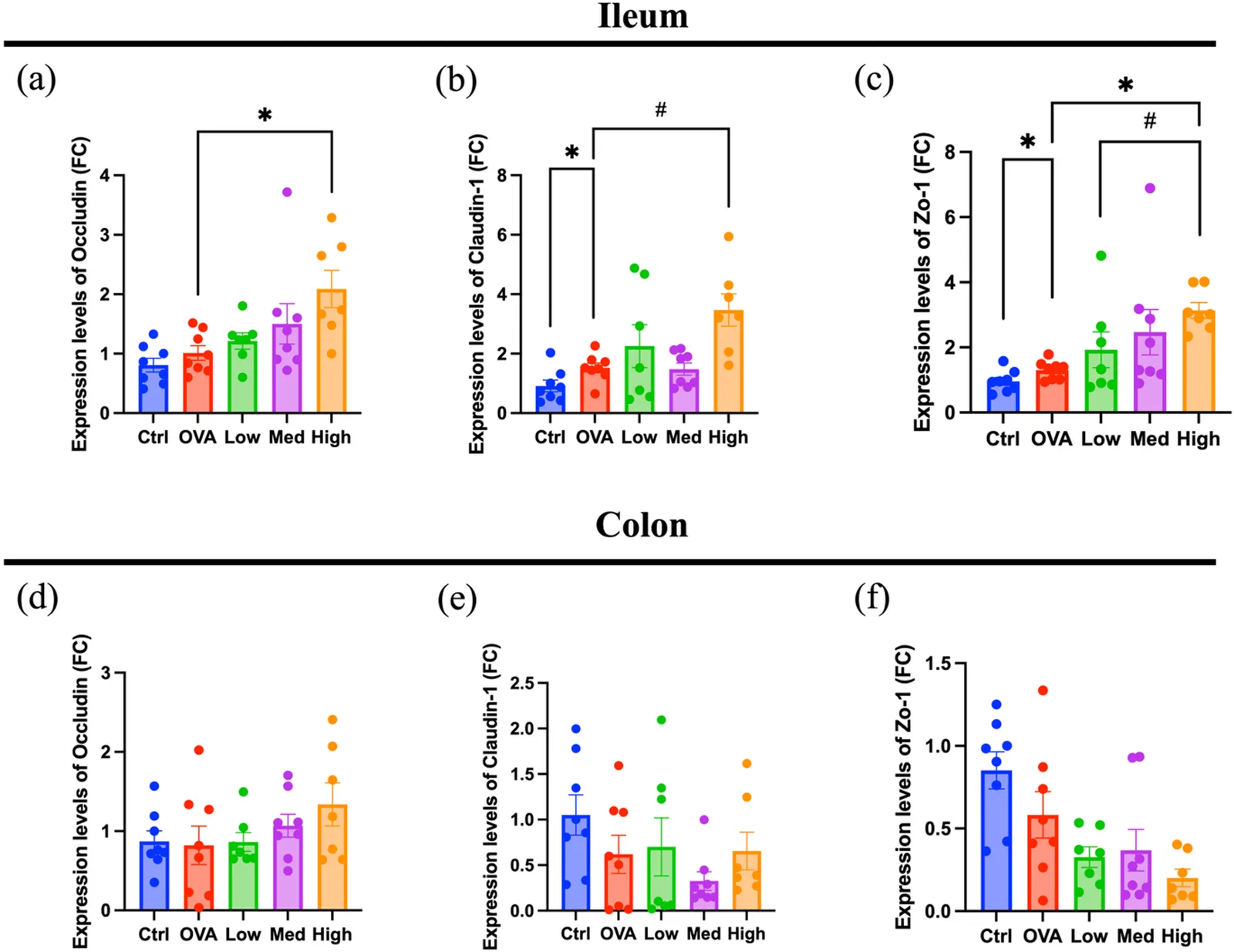
Gene expression levels of tight junction-related genes in the ileum and colon following FM8 supplementation. (**a**–**f**) Expression levels of (**a**,** d**) *Occludin*, (**b**,** e**) *Claudin-1*, and (**c**,** f**) *Zo-1* in ileum (**a**–**c**) and (**d**–**f**) colon. Plots represent individual mice, and bars represent mean ± standard error of the mean. #*p* < 0.1, *p < 0.05 vs. ovalbumin (OVA) group (Mann–Whitney U test or Kruskal–Wallis test followed by Dunn’s multiple comparisons test). Control (Ctrl): non-allergy group, no intervention. OVA: OVA-induced allergy group, no intervention. Low: OVA allergy group administered FM8 at a low dose. Med: OVA allergy group administered FM8 at a medium dose. High: OVA allergy group administered FM8 at a high dose

Quantification of the expression levels of tight junction-associated genes in the ileum showed significant upregulation of *Claudin-1* and *Zo-1* in the OVA group compared with that in the Ctrl group (*p* = 0.0329 and 0.0329, respectively), whereas *Occludin* expression showed no significant difference (*p* = 0.2786). In contrast, compared with the OVA group, the High group showed significant upregulation of *Occludin* and *Zo-1* and an increasing trend in *Claudin-1* expression (*Occludin*: *p* = 0.0195; *Claudin-1*: *p* = 0.0840, increasing trend; *Zo-1*: *p* = 0.0316). A modest trend was also observed between the Med- and High groups for *Zo-1* expression (*p* = 0.0909; Fig. 4a–c). These findings suggest that FM8, particularly at the high dose, may exert a barrier-restorative effect in the ileum. However, no significant changes in the expression of these genes were observed in the colon (*Occludin*: *p* = 0.9999; *Claudin-1*: *p* = 0.9999; *Zo-1*: *p* = 0.1009), indicating that the effects of FM8 on tight junction regulation may be region-specific (Fig. 4d–f).

**Fig. 5:**
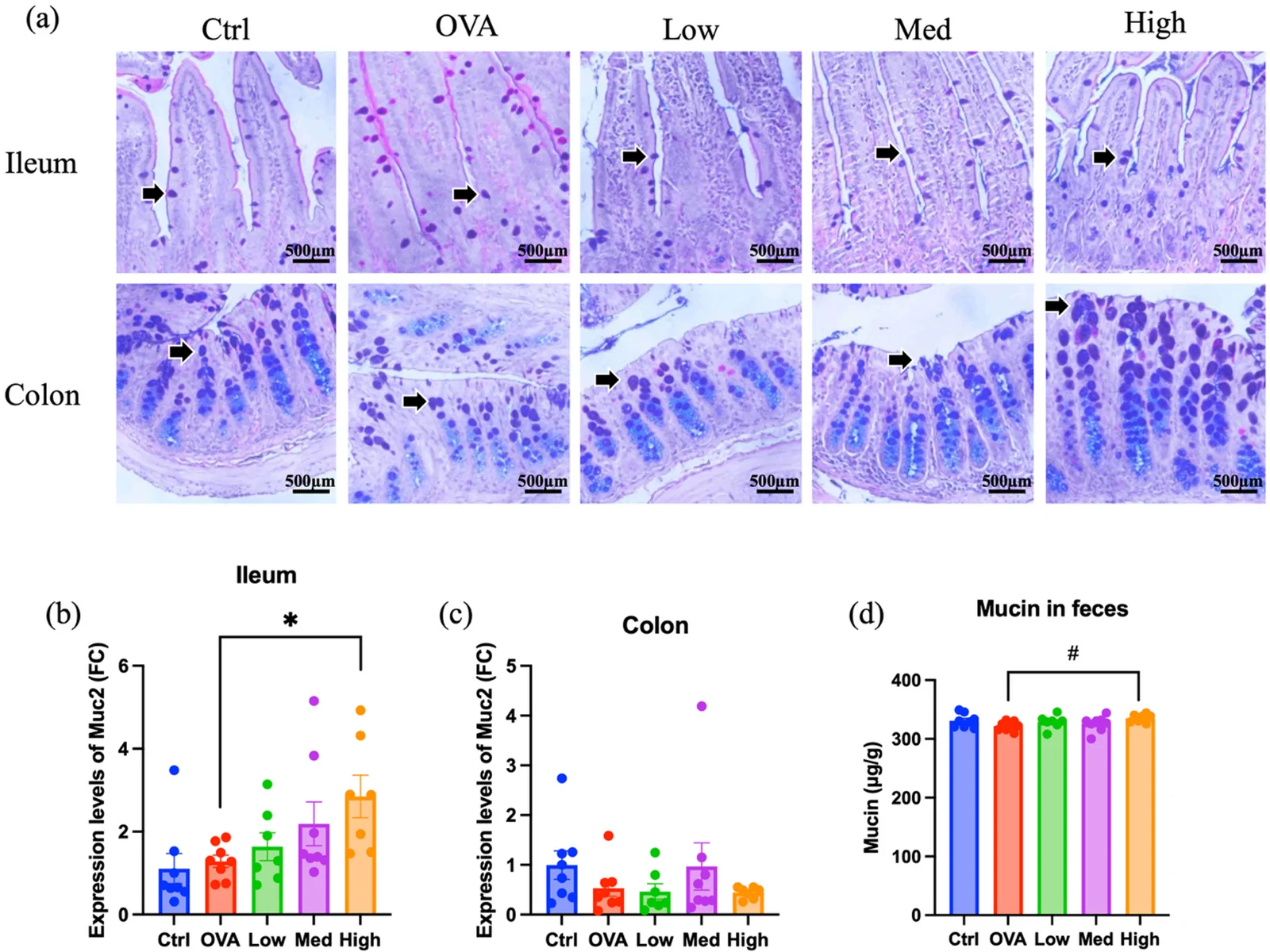
Alcian blue-Periodic acid-Schiff (AB-PAS) staining and *Muc2* expression in the ileum, colon, and fecal mucin levels following FM8 supplementation. (**a**) AB-PAS staining of the ileum and colon. (**b**,** c**) *Muc2* expression levels in the (**b**) ileum and (**c**) colon. (**d**) Fecal mucin levels. Goblet cell production (arrows) could be observed at every segment. Plots represent individual mice, and bars represent mean ± standard error of the mean. #*p* < 0.1, *p < 0.05 vs. ovalbumin (OVA) group (Mann–Whitney U test or Kruskal–Wallis test followed by Dunn’s multiple comparisons test). Control (Ctrl): non-allergy group, no intervention. OVA: OVA-induced allergy group, no intervention. Low: OVA allergy group administered FM8 at a low dose. Med: OVA allergy group administered FM8 at a medium dose. High: OVA allergy group administered FM8 at a high dose

Subsequently, histological analysis of the ileum and colon was performed using Alcian blue-Periodic acid-Schiff (AB-PAS) staining, which differentiates neutral mucins (magenta, PAS), acidic mucins (blue, Alcian blue), and mixed mucins (purple). All groups showed magenta and purple staining, with more intense coloration observed in the High group, indicating increased mucin production (Fig. 5a). Furthermore, *Muc2* expression was significantly elevated in the High group compared with that in the OVA group in the ileum (*p* = 0.0374), whereas no significant differences were observed in the colon (*p* = 0.9999; Fig. 5b, c). Consistent with these findings, fecal mucin content showed an increasing trend in the High group (*p* = 0.0697), suggesting the potential of FM8 to enhance mucosal barrier function (Fig. 5d).

### Effects of FM8 on the intestinal microbiota

**Fig. 6:**
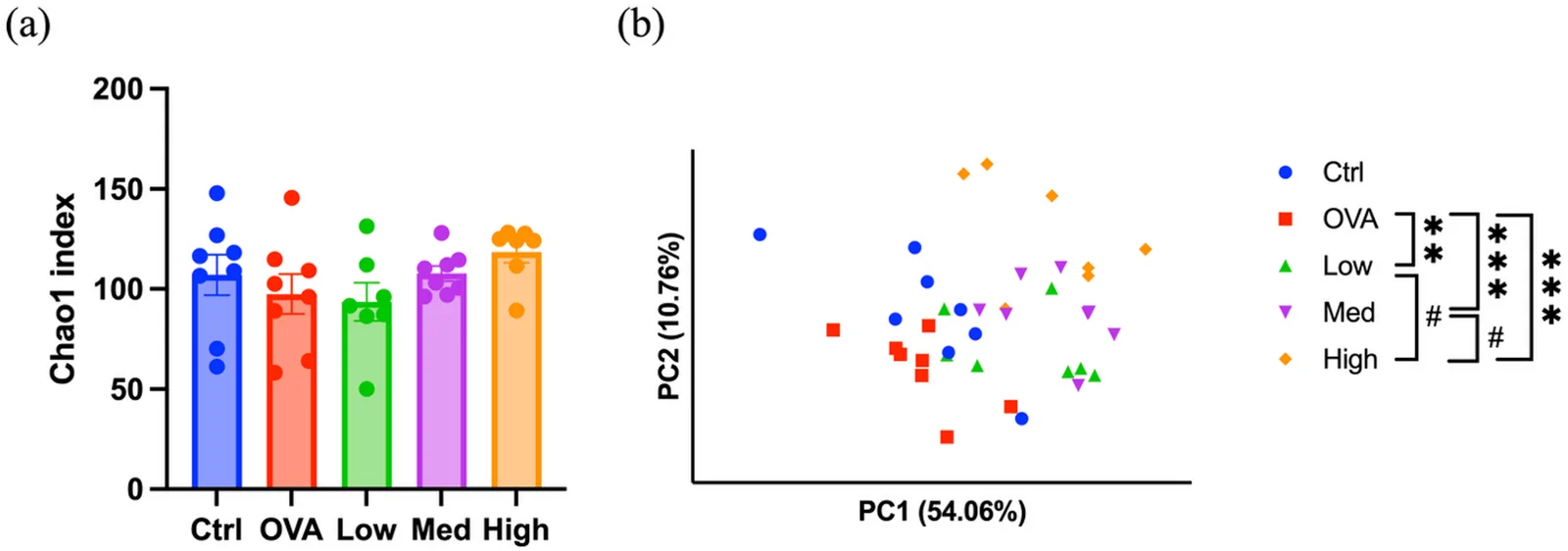
Alpha and beta diversity of intestinal microbiota in each experimental group. (**a**) Chao1 index values (alpha diversity). (**b**) Principal coordinate analysis based on weighted UniFrac distances (beta diversity). The first principal component (PC1) is plotted on the x-axis and the second principal component (PC2) on the y-axis. Plots represent individual mice, and bars represent mean ± standard error of the mean. #*p* < 0.1, *p < 0.05 vs. OVA group. Control (Ctrl): non-allergy group, no intervention. Ovalbumin (OVA): OVA-induced allergy group, no intervention. Low: OVA allergy group administered FM8 at a low dose. Med: OVA allergy group administered FM8 at a medium dose. High: OVA allergy group administered FM8 at a high dose

Next, we investigated the effects of FM8 intake on gut microbiota composition. Assessment of the taxonomic diversity based on the Chao1 index (an indicator of α-diversity) showed no significant differences among the groups (Fig. 6a).

PCoA and ecological distance analysis using weighted UniFrac distances (an indicator of β-diversity) also showed no significant differences in β-diversity between the Ctrl and OVA groups (*p* = 0.371; Fig. 6b). However, β-diversity between the OVA and FM8 intake groups differed (*p* = 0.008, Low; *p* = 0.001, Med; and *p* = 0.001, High). Additionally, no significant differences were observed between the Low- and Med groups (*p* = 0.280), whereas the High group exhibited a trend toward differentiation from both the Low- and Med groups (*p* = 0.054 and 0.060, respectively).

**Fig. 7:**
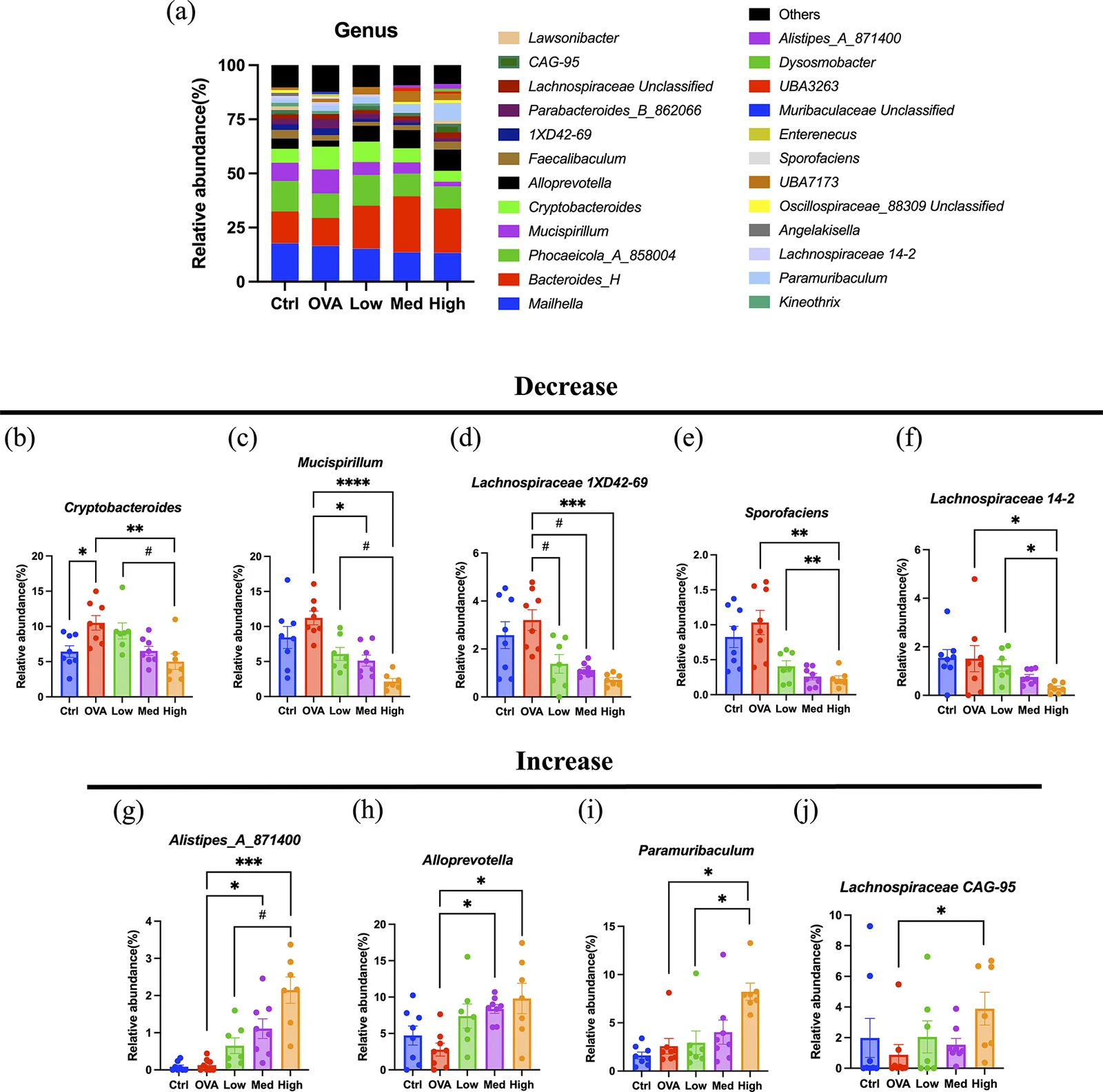
Relative abundance of gut microbiota at the genus level following FM8 supplementation. (**a**) Taxa bar plots showing relative abundance across experimental groups. (**b**–**f**) Relative abundance of genera significantly decreased in the High group in different groups: (**b**) *Cryptobacteroides*, (**c**) *Mucispirillum*, (**d**) *Lachnospiraceae 1XD42-69*, (**e**) *Lachnospiraceae 14−2*, (**f**) *Sporofaciens*. (**g**–**j**) Relative abundance of genera significantly increased in the High group in different groups: (**g**) *Alistipes_A_871400*, (**h**) *Alloprevotella*, (**i**) *Paramuribaculum*, (**j**) *Lachnospiraceae CAG-95*. Plots represent individual mice, and bars represent mean ± standard error of the mean. #*p* < 0.1, *p < 0.05, **p < 0.01, ***p < 0.001, ****p < 0.0001 vs. ovalbumin (OVA) group (Mann–Whitney U test or Kruskal–Wallis test followed by Dunn’s multiple comparisons test). Control (Ctrl): non-allergy group, no intervention. OVA: OVA-induced allergy group, no intervention. Low: OVA allergy group administered FM8 at a low dose. Med: OVA allergy group administered FM8 at a medium dose. High: OVA allergy group administered FM8 at a high dose

To further investigate specific microbial changes induced by FM8, we analyzed the relative abundance of genera with > 1% relative abundance, visualized using taxa bar plots (Fig. 7a). Among the examined genera, *Cryptobacteroides* was significantly increased in the OVA group compared with that in the Ctrl group (*p* = 0.0148), suggesting a potential association with allergic inflammation. Moreover, its abundance was significantly reduced in the High group compared with the OVA group (*p* = 0.0107), with a trend toward reduction compared with the Low group (*p* = 0.0816; Fig. 7b).

Based on the observed effects of FM8 on IL-10 induction and allergy suppression, targeted comparisons between the OVA- and High groups revealed several significantly altered genera. The relative abundances of *Mucispirillum*, *Lachnospiraceae 1XD42-69*, *Lachnospiraceae 14−2*, and *Sporofaciens* were significantly decreased in the High group compared with those in the OVA group (*Mucispirillum*: *p* < 0.0001; *Lachnospiraceae 1XD42-69*: *p* = 0.0005; *Lachnospiraceae 14−2*: *p* = 0.0345; *Sporofaciens*: *p* = 0.0031). Intermediate reductions were also observed in Med- or Low groups for certain genera (e.g., *Mucispirillum*: OVA vs. Med, *p* = 0.0323; *Sporofaciens*: OVA vs. Med, *p* = 0.0072).

Conversely, several genera were significantly enriched in the High group, including *Alistipes_A_871400* (OVA vs. High, *p* = 0.0002), *Alloprevotella* (*p* = 0.0197), *Paramuribaculum* (*p* = 0.0174), and *Lachnospiraceae CAG-95* (*p* = 0.0467). In many cases, differences between the High- and Low groups approached significance, suggesting potential dose-dependent effects (e.g., *Alistipes_A_871400*: Low vs. High, *p* = 0.0829; *Paramuribaculum*: *p* = 0.0313; *Lachnospiraceae CAG-95*: *p* = 0.0313; Fig. 7c–j).

### Effects of FM8 on SCFAs and lactate concentrations in cecal contents

Commensal bacteria metabolize dietary fiber to produce SCFAs, which are critical for Treg cell expansion and promotion of immunosuppressive functions such as IL-10 production, thereby regulating proinflammatory responses in the gut [38].

**Fig. 8:**
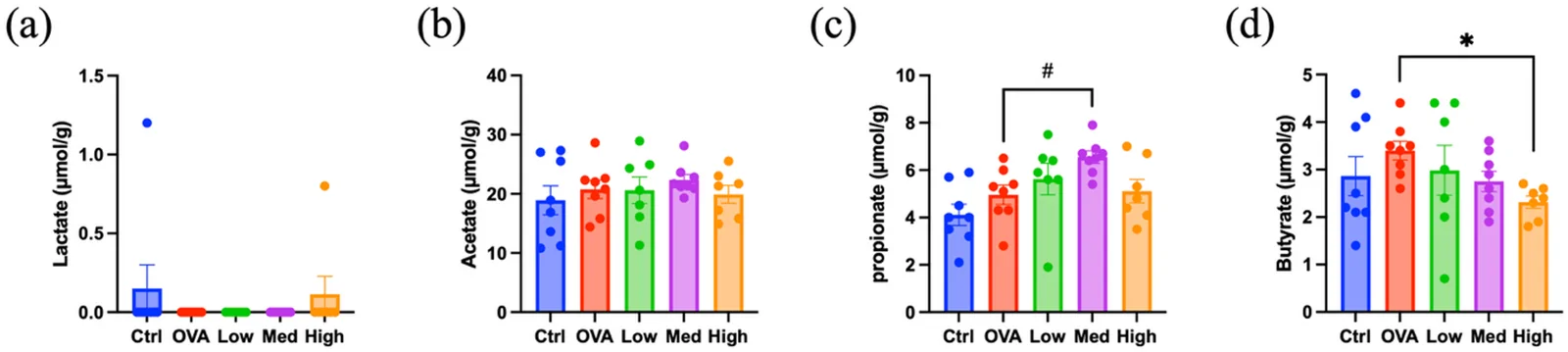
Changes in short-chain fatty acids (SCFAs) and lactate levels in cecal samples following FM8 supplementation (**a**–**d**) Changes in (**a**) acetate, (**b**) propionate, (**c**) n-butyrate, and (**d**) lactate levels in cecal samples of ovalbumin (OVA)-induced allergic mice with and without FM8 administration. Plots represent individual mice, and bars represent mean ± standard error of the mean. #*p* < 0.1, *p < 0.05 vs. OVA group (Mann–Whitney U test or Kruskal–Wallis test followed by Dunn’s multiple comparisons test). Control (Ctrl): non-allergy group, no intervention. OVA: OVA-induced allergy group, no intervention. Low: OVA allergy group administered FM8 at a low dose. Med: OVA allergy group administered FM8 at a medium dose. High: OVA allergy group administered FM8 at a high dose

Therefore, we assessed the effect of FM8 on SCFAs and lactate concentrations by measuring the concentrations of major SCFAs and lactate in cecal contents. The findings revealed an increasing trend for propionate levels in the Med group compared with those in the OVA group (*p* = 0.0511). In contrast, butyrate levels were significantly reduced in the High group compared with those in the OVA group (*p* = 0.0270; Fig. 8).

## Discussion

Previous studies have suggested the anti-inflammatory potential of FM8. However, its anti-allergic effects have not been reported to date. This study focused on the close relationship between the gut microbiota and immune function and investigated the preventive effects of FM8 on allergic responses and gut homeostasis using an OVA-induced food allergy model.

The OVA group showed typical allergy markers, including increased symptom scores, decreased rectal temperature, elevated serum OVA-sIgE levels, and upregulated *IL-4* mRNA expression in Peyer’s patches (Fig. 2). In contrast, FM8 supplementation suppressed these markers in a dose-dependent manner, suggesting that FM8 exerts a preventive effect against food allergy.

Previous in vitro studies have shown that FM8 strongly promotes IL-10 production [23]. Therefore, we hypothesized that the preventive effects of FM8 against food allergy are mediated by IL-10 induction. To test this hypothesis, we measured *IL-10* mRNA expression in intestinal tissues and IL-10 protein in serum. FM8 supplementation increased *IL-10* mRNA expression in Peyer’s patches, ileum, and colon, with a significant elevation in serum IL-10 levels in the High group. As IL-10 suppresses Th2 cytokines [9, 10] and induces Th2 cell apoptosis [39], these findings suggest that FM8 may prevent allergic responses, at least in part, by inducing IL-10 and thereby attenuating Th2-associated responses, including OVA-specific IgE production. However, Th1-associated cytokines were not quantified in this study. Because food allergy is characterized by a Th2-skewed immune imbalance, the absence of Th1 profiling limits the ability to assess the effect of FM8 on overall Th1/Th2 immune balance. Future studies should include measurements of Th1-associated cytokines and broader antigen-specific T-cell profiling to clarify how FM8 modulates the immune response.

Given these IL-10-related findings, we next focused on intestinal barrier-related responses, because IL-10 is known to enhance barrier integrity by acting on epithelial and immune cells to strengthen tight junctions and reduce intestinal permeability [40]. We therefore examined the expression of tight junction-associated genes (*Occludin*, *Claudin-1*, and *Zo-1*) in intestinal tissues. In the High group, ileal expression of these genes was increased, with significant upregulation of *Occludin* and *Zo-1* and a trend toward increased *Claudin-1* expression, suggesting enhanced barrier-related responses; however, no changes were observed in the colon (Fig. 4). We also examined mucin-related responses, as the mucus layer represents another important component of the intestinal barrier. The High group exhibited increased acidic mucopolysaccharide staining, significant upregulation of ileal *Muc2* expression, and a trend toward higher fecal mucin levels, whereas colonic *Muc2* expression was unchanged (Fig. 5). Hasnain et al. reported that IL-10 supports mucus barrier production by suppressing MUC2 misfolding and endoplasmic reticulum stress in goblet cells [41]. Similarly, studies using IL-10-deficient mice have shown a significant reduction in *Muc2* expression [42]. Thus, the increases in *Muc2* expression and fecal mucin levels observed in this study may be associated with enhanced IL-10 production induced by FM8. Taken together, these tight junction- and mucin-related changes suggest that FM8 may enhance intestinal barrier-related responses, particularly in the ileum. This ileum-predominant pattern likely reflects regional differences in intestinal physiology, with the ileum representing a primary site of allergen exposure and absorption, whereas the colon is a major site of microbial fermentation. Accordingly, FM8 may preferentially modulate barrier-related responses at the site of allergic inflammation in this model. Koida et al. [32] reported that enhancing barrier function suppresses allergen absorption, suggesting that the observed gene expression and mucin-related changes may contribute to reduced allergen uptake through enhanced gut barrier-related responses.

Probiotics and prebiotics are widely known to influence gut microbiota composition and contribute to host immune function [43]. Therefore, in this study, we assessed whether FM8-induced IL-10 production and immunomodulatory effects are associated with alterations in the gut microbiota. 16S rRNA amplicon sequencing of cecal samples showed a notable increase in the relative abundance of *Cryptobacteroides* in the OVA group, which was reduced in the High group. Although the functional relevance of *Cryptobacteroides* is not established, previous studies have reported that *Cryptobacteroides* increases following exposure to perfluorolauric acid, a persistent organic pollutant [44], whereas it decreases in a diabetic constipation model after therapeutic intervention [45]. In addition, a treatment study using *Lacticaseibacillus* combined with skim milk in a mouse model of cognitive impairment reported a reduction in *Cryptobacteroides* and a negative correlation with cognitive performance indices [46]. Taken together, these reports and our observation suggest that *Cryptobacteroides* may be associated with inflammatory or dysbiotic conditions. However, the mechanisms by which it may contribute to inflammation, including in allergic disease, as well as its metabolic outputs, remain unclear. Therefore, further studies using functional approaches, including metagenomics and metabolomics, and validation in relevant allergy models will be required to clarify the role of this taxon. The High group also showed decreased relative abundances of *Mucispirillum*, *Lachnospiraceae 1XD42-69*,* Lachnospiraceae 14−2*, and *Sporofaciens*, along with increased abundances of *Alistipes_A_871400*,* Alloprevotella*,* Paramuribaculum*, and *Lachnospiraceae CAG-95*. Several of the taxa enriched in the High group have been reported to have SCFA-producing potential [47–49]. SCFAs are important microbiota-derived metabolites involved in immune regulation and intestinal barrier maintenance. Previous studies have demonstrated that probiotics and prebiotics can elevate intestinal SCFA concentrations [50, 51], and children with food allergies have been reported to exhibit lower fecal SCFA concentrations [52]. SCFA-related interventions have been shown to alleviate allergic responses in mouse models, and high fecal butyrate and propionate levels in early life have been associated with a lower risk of atopy in humans [53, 54]. Mechanistically, SCFAs act through G protein-coupled receptors, such as GPR43 and GPR81, on immune cells and modulate dendritic cells, T cells, and macrophages. Acetate, propionate, and butyrate enhance IL-10 expression in regulatory Th1 cells via GPR43 activation [55, 56], while lactate activates GPR81 [57]. Moreover, SCFAs are known to restore tight junction integrity and increase mucin secretion [38]. Based on these reports and the observed enrichment of taxa with reported SCFA-producing potential, we measured cecal SCFA concentrations to determine whether FM8-induced immune and barrier-related changes were accompanied by increased SCFA production.

In the present study, however, cecal SCFA concentrations did not increase following FM8 administration; notably, butyrate levels were significantly decreased in the FM8-treated groups. Thus, enrichment of taxa with reported SCFA-producing potential did not translate into higher measurable cecal SCFA concentrations in this study. Two possible explanations may account for this observation. First, cecal SCFA concentrations reflect not only microbial production but also host- and microbe-driven utilization and absorption. Because butyrate serves as a major energy source for intestinal epithelial cells [58, 59], decreased cecal butyrate may reflect increased epithelial uptake and utilization during barrier remodeling. This interpretation is consistent with the upregulation of mucin- and tight junction-related genes observed in the ileum. Second, FM8 is a paraprobiotic composed of non-viable bacterial cells and therefore cannot directly produce SCFAs in the same way as metabolically active probiotics. Instead, FM8 may promote IL-10 production through host immune recognition of bacterial cell components. Previous studies have reported that bacterial cell wall components can stimulate Toll-like receptors and other pattern recognition receptors, leading to IL-10 production [60, 61]. Through such SCFA-independent IL-10 induction, FM8 may indirectly modulate the gut ecosystem and support mucin-related barrier responses. Future studies should clarify the relationship among FM8-induced changes in gut microbiota, SCFA production and utilization, IL-10 induction, and barrier-related responses.

This study has some limitations.

First, although FM8 showed preventive effects in this murine OVA-induced food allergy model, it remains unclear whether similar effects would be observed in humans, especially at the highest dose tested. Using standard body-surface-area scaling and the average mouse body weight in this study, the high dose corresponds to approximately 1.05 × 10^13^ CFU-equivalents/day for a 60-kg adult [62]. This is higher than doses used in many human studies of live probiotics [63, 64]. However, FM8 is heat-killed, so this value should be considered only as a rough guide. In addition, reported doses of paraprobiotics vary widely across studies, and units are inconsistent [17, 65]. Therefore, it is still unclear what dose is appropriate for humans. Future studies should investigate the effective dose of FM8 in humans.

Second, this study was conducted using an OVA-induced murine model of food allergy; therefore, only OVA was used as the allergen, and the effects of FM8 on other clinically relevant food allergens, such as peanut or milk proteins, have not been examined. Given potential allergen-specific differences in immune responses, future studies should evaluate the efficacy of FM8 in models using various food allergens.

Third, the specific immune cell populations responsible for FM8-induced IL-10 production were not identified. IL-10 is produced by multiple immune cell types, including Tregs, dendritic cells, and macrophages [66]. However, this study did not determine the cellular sources of FM8-induced IL-10. Detailed analyses using flow cytometry or single-cell RNA sequencing will be necessary to clarify the immune cell subsets involved.

Last, while FM8 was suggested to enhance gut barrier function, our evidence is based primarily on transcriptional changes and mucin-related readouts, and direct functional measurements of barrier integrity were not performed. Because tight junction mRNA expression does not necessarily correlate with protein abundance or junctional assembly, future studies should include protein-level validation of tight junction components, such as immunoblotting or immunostaining for *Occludin*, *Claudin-1*, and *Zo-1*, as well as functional permeability assays such as fluorescein isothiocyanate–dextran, to confirm barrier improvement.

## Conclusion

The paraprobiotic *Lactiplantibacillus plantarum* FM8 attenuates food allergy–associated responses in a murine model, accompanied by increased IL-10 and ileal barrier-related responses, as well as shifts in gut microbiota composition. Notably, these effects were not accompanied by an increase in cecal SCFA concentrations. These findings provide novel insights into the mechanisms of paraprobiotics in immune modulation. However, further clinical studies are necessary to confirm the efficacy and safety of FM8 in humans and evaluate its potential as a preventive or therapeutic strategy for food allergies.

## Supplementary Information

Below is the link to the electronic supplementary material.

Supplementary Material 1

## Data Availability

All data reported in this study are included with this manuscript.The 16S rRNA gene NGS datasets were deposited in The NCBI Sequence Read Archive (SRA) (accession number: PRJNA1298223).

## Abbreviations

Th2: T helper type 2
IgE: Immunoglobulin E
IL-10: Interleukin-10
Treg: Regulatory T
OVA: Ovalbumin
PBS: Phosphate-buffered saline
OVA-sIgE: OVA-specific IgE
RT-qPCR: Quantitative reverse transcription PCR
PAS: Periodic acid–Schiff
AB: Alcian blue
PCoA: Principal coordinate analysis
SCFAs: Short-chain fatty acids
HPLC: High-performance liquid chromatography
