# Supplementary material for: Preventive effect of heat-killed Lactiplantibacillus plantarum FM8 in an ovalbumin-induced food allergy murine model
Source: Allergy Asthma Clin Immunol. 2026 Jun 25;22:48. doi: 10.1186/s13223-026-01048-8 (PMC13555864; doi:10.1186/s13223-026-01048-8)
Supplement: Supplementary file 1 [file 13223_2026_1048_MOESM1_ESM.docx]

**Supplementary Tables**

**Table S1.** PCR primers used in the present study.

| **Target gene** | **Primer name** | **Oligonucleotide sequence** | **Reference** |
| --- | --- | --- | --- |
| 18S rRNA | *18S rRNA* forward | TTGGAGGGCAAGTCTGGTG | [29] |
|  | *18S rRNA* reverse | CCGCTCCCAAGATCCAACTA |  |
| *IL-4* | *Il4*forward | GGCATTTTGAACGAGGTCAC | [30] |
|  | *Il4*reverse | AAATATGCGAAGCACCTTGG |  |
| *IL-10* | *Il10*forward | TGAATTCCCTGGGTGAGAAG | [30] |
|  | *Il10*reverse | TGGCCTTGTAGACACCTTGG |  |
| *Muc2* | *Muc2* forward | TGCTGACGAGTGGTTGGTGAATG | [31] |
|  | *Muc2* reverse | TGATGAGGTGGCAGACAGGAGAC |  |
| *Occludin* | Occludin forward | TGGCTATGGAGGCGGCTATGG | [31] |
|  | Occludin reverse | AAGGAAGCGATGAAGCAGAAGGC |  |
| *Claudin-1* | Claudin-1 forward | GCTGGGTTTCATCCTGGCTTCTC | [31] |
|  | Claudin-1 reverse | CCTGAGCGGTCACGATGTTGTC |  |
| *Zo-1* | *Zo1*forward | GCGAACAGAAGGAGCGAGAAGAG | [31] |
|  | *Zo1*reverse | GCTTTGCGGGCTGACTGGAG |  |

Table S2. Basic characteristics of animals during the rearing period.

|  | Ctrl | | | OVA | | | Low | | | Med | | | High | | | Statistical significance |
| --- | --- | --- | --- | --- | --- | --- | --- | --- | --- | --- | --- | --- | --- | --- | --- | --- |
| Body weight change (g) | 4.99 | ± | 0.20 | 5.14 | ± | 0.27 | 4.94 | ± | 0.38 | 5.73 | ± | 0.17 | 5.07 | ± | 0.24 | n.s. |
| Feed intake (g/day) | 2.81 | ± | 0.06 | 2.84 | ± | 0.05 | 2.67 | ± | 0.05 | 2.84 | ± | 0.03 | 2.75 | ± | 0.03 | n.s. |
| FM8 intake (1.0 × 10^8^ CFU/day) |  | - |  |  | - |  | 21.38 | ± | 0.17 | 113.67 | ± | 0.48 | 549.68 | ± | 2.33 |  |
| Cecal weight/body weight (g/g) | 0.01 | ± | 0.00 | 0.01 | ± | 0.00 | 0.01 | ± | 0.00 | 0.01 | ± | 0.00 | 0.01 | ± | 0.00 | n.s. |
| Cecal contents weight/body weight (g/g) | 0.00 | ± | 0.00 | 0.01 | ± | 0.00 | 0.01 | ± | 0.00 | 0.01 | ± | 0.00 | 0.01 | ± | 0.00 | n.s. |
| OVA-specific IgE at baseline | 0.01 | ± | 0.00 | 0.01 | ± | 0.00 | 0.02 | ± | 0.00 | 0.01 | ± | 0.00 | 0.01 | ± | 0.00 | n.s. |

Values are expressed as mean ± SEM. n.s., not significant.
